# Subthreshold violations of trajectory predictions are sensitive to TMS of cerebellum Crus I/II

**DOI:** 10.1016/j.ynirp.2026.100382

**Published:** 2026-07-20

**Authors:** Ellen Joos, Camille Scherer, Philippe Isope, Jack Foucher, Anne Giersch

**Affiliations:** a University of Strasbourg, INSERM U1329 Strasbourg Translational nEeuroscience and Psychiatry (STEP) team Psychiatry, University Hospital of Strasbourg, ITI Neurostra, France; b Institute for Frontier Areas of Psychology and Mental Health (IGPP), Freiburg, Germany; c Department of Psychiatry and Psychotherapy, Medical Center, University of Freiburg, Freiburg, Germany; d Faculty of Medicine, University of Freiburg, Freiburg, Germany; e Institut des Neurosciences Cellulaires et Intégratives, Centre National de la Recherche Scientifique, Université de Strasbourg, Strasbourg, 67084, France; f ICube - CNRS UMR 7357, Neurophysiology, FMTS, University of Strasbourg, France; g CEMNIS - Noninvasive Neuromodulation Center, University Hospital Strasbourg, France

**Keywords:** Illusion, Motion regularity, Trajectory irregularity, Prediction error, Modulation, TMS, EEG

## Abstract

We recently suggested that when visual objects move towards each other, it may involve a trajectory prediction and sensory checking at the level of milliseconds. We examined this possibility by exploring the consequences of a sub-threshold manipulation of squares trajectories on a newly found illusion, i.e., the perception of a gap at the time of the collision. We first examined the consequences of the manipulation on the conscious illusion. In a more exploratory way, we studied the EEG correlates of the manipulation, as well as the impact of transcranial magnetic stimulation (TMS) on the cerebellum (right Crus I/II). The TMS was a typical intermittent theta-burst stimulation, but only one sequence of around 3 min, compared with a placebo stimulation. All participants participated to two TMS sessions, in the sham-verum or in the verum-sham order. The trajectory manipulation occurred within less than 60 ms before the collision and reliably decreased the illusion rate. The results suggest that the prediction is temporarily stopped after the trajectory change. The illusion was accompanied by late positive ERP signals in CP4. The amplitude of these signals was modulated by TMS verum on the cerebellum, but only in the sham-verum order. As a whole the results are consistent with automatic verifications of the trajectory regularity at the millisecond level.

## Highlights

• As In earlier results, two colliding squares elicit the illusory perception of a space.
• The illusion decreases in case of a sub-threshold irregularity of the squares' trajectory.
• The effect of the trajectory manipulation is accompanied by a late positive EEG signal.
• EEG amplitudes are to some extent affected by intermittent theta-burst transcranial magnetic stimulation on the cerebellum.

## 1 Introduction

When two objects move towards each other we usually predict a collision. This prediction relies on statistical regularities and the anticipation of the future position of the objects. It stands to reason that given the need to avoid a collision, the precision of the prediction is important. Here we explore if this is true at the level of milliseconds.

Current theories postulate that the brain detects regularities in the sensory input and forms predictions accordingly in an internal model (Wolpert et al., 1998). A difference between the model and the actual sensory information leads to so-called “prediction errors”, which signals the need to adjust behavior (Giersch et al., 2016; Shadmehr et al., 2010; Sokolov et al., 2017). However, what makes a prediction error pertinent or not is not always straightforward. In everyday life, it is frequently the case that there are unexpected glitches, even when events unfold as usual, but those glitches are sometimes best ignored. For example, it is unclear if subthreshold irregularities in a car trajectory in front of us are or not significant (subthreshold irregularities means irregularities that are consciously perceived in less than 50% of the cases). It has already been shown that subthreshold prediction errors are processed (Charles et al., 2013, 2014; Rowe et al., 2020), but their impact on perception and whether or not their processing is modulated is unclear. What happens if the prediction error has been processed in the brain, e.g., the irregularity in the car trajectory in front of us, but we are not aware of this irregularity? This question differs from the exploration on reward prediction error, saliency, or sensory mismatch. There is no manipulation of reward involved in our task, and we do not expect any link with the reward pathways. We neither expect that the perturbation will attract attention, or that saliency and sensory mismatch pathways will be involved, for the following reasons. Predictive coding has been used to explain how attention can be attracted towards an unexpected information, thus leading to the concept of saliency (Friston, 2009). Predictive coding also explains sensory mismatch, when an irregularity arises within a train of regular sensory information, leading to mismatch negativity on electroencephalographic (EEG) recordings (Friston, 2005). Those EEG signals, as well as saliency, are usually observed when the irregularity is not only rare but emerges at the conscious level (Flynn et al., 2017). In our task however, there is no conscious realisation of the trajectory manipulation (see supplementary material S1), which happens in half of the trials, i.e., more frequently than in tasks yielding mismatch negativity. Here we explore if the trajectory prediction includes a prediction at the level of milliseconds. If it does not, we do not expect any impact of the trajectory manipulation, whether on behavior or EEG. If it does, sub-threshold irregularities should impact the prediction of a movement trajectory and its effect on conscious perception. In a more exploratory way, we examine whether it affects EEG signals and whether the processing of irregularities can be modulated by a short stimulation on the cerebellum, given the growing literature showing its involvement in temporal prediction and millisecond-level processes (Sokolov et al., 2017).

### 1.1 The role of cerebellum

The cerebellum is an obvious target for our goal (Giersch et al., 2016; Parker, 2016; Picard et al., 2008), and this for several reasons. First, even though the cerebellum is mainly known for its role in motor functions and in motor predictions (Paulin, 1993; Person, 2019), its involvement in sensory and cognitive mechanisms is now largely recognized (Andreasen et al., 1999; Argyropoulos et al., 2020; Leiner et al., 1993; Schmahmann, 2010), especially its role in the general formation of predictions (Popa and Ebner, 2019; Sokolov et al., 2017). It is not the only area involved in the processing of prediction errors (Alexander and Brown, 2019; Man et al., 2024), but it has been shown to code prediction errors in case of motion, like e.g., unexpected self-motion (Brooks and Cullen, 2013). Most importantly, it is also known to be involved in time processing at the level of milliseconds (Coull et al., 2011; Ivry and Spencer, 2004), which makes it a choice target when considering sub-threshold trajectory manipulations. The cerebellum, and especially its lateral part (Crus I, Lobule VII) is involved in the detection of motion and the prediction of temporo-spatial information in the case of visual trajectories, both in humans (Baumann et al., 2015; Baumann and Mattingley, 2010; O'Reilly et al., 2008) and cats (Cerminara et al., 2009). For example, based on the current state of the body and motor commands originating in the cerebral cortex, cerebellar internal models are updated through the modification of synaptic transmission between granule cells and Purkinje cells (Cullen, 2023; Spaeth et al., 2022; Streng et al., 2022; Kimpo et al., 2014; Wolpert et al., 1998). This plasticity is controlled by the climbing fiber pathway as it conveys error and/or reward signals to the Purkinje cell (Hull, 2020). Error predictions can therefore be encoded by Purkinje cell discharge, which is transmitted to the cerebral cortex via the cerebellar nuclei and the thalamus (Li and Mrsic-Flogel, 2020; Ramnani, 2006). For these reasons, an impact of the stimulation on the cerebellum is expected on the processing of trajectory manipulations at the level of milliseconds, and a confirmation of such an impact would in turn reinforce the idea of the involvement of millisecond-level prediction errors in the task. Besides TMS is expected to affect neuronal plasticity (Jannati et al., 2023), and may thus modulate the processing of such prediction errors.

### 1.2 The impact of TMS on the cerebellum, and the rationale for the choice of stimulation

Non-invasive stimulation of the cerebellum is increasingly used to modulate cerebellar-cortical network activity. The anatomical location of the cerebellum, its high responsiveness of the cerebellar cortex to stimulation, and the involvement of the cerebellum in numerous cerebellar-cortical networks make the cerebellum an ideal target for investigation (van Dun et al., 2018). The application of a magnetic field on the skull induces local electric currents within the brain (Jannati et al., 2023). Cerebellar stimulation activates Purkinje cells via parallel fibers, which in turn inhibit the deep cerebellar nuclei, which have excitatory connections with the cortex via the thalamus (Spampinato et al., 2024). Several studies have explored the impact of TMS (Transcranial Magnetic Stimulation) targeting the cerebellum (Mioni et al., 2020) and suggest a role of the cerebellum in the processing of moving object trajectories. For example, cerebellar patients are impaired at discriminating the velocity of moving objects (Ivry and Diener, 1991) or at discriminating motion from noise (Thier et al., 1999). Nonetheless, stimulating the Cerebellum with TMS is difficult due to the depth of the structure. Also, several potential side effects have been described in humans. As a matter of fact, stimulating the cerebellum is reputed to be difficult due to its proximity to the neck muscles and to the vestibular system, potentially inducing muscle contractures, dizziness or nausea (Hurtado-Puerto et al., 2020; Satow et al., 2002). Hurtado-Puerto et al. (2020) showed that theta-burst stimulations were well tolerated, with around 4% of side-effects occurrences. For these reasons we have been using a short theta-burst stimulation. The duration of our TMS was rather short (Jannati et al., 2023), which necessarily limited our chance of seeing an impact of TMS. The duration of the effect with 600 pulses is expected to decrease after 1 h (Wischnewski and Schutter, 2015; Chung et al., 2016) albeit there is a lack of knowledge regarding the duration of the impact of cerebellum stimulation specifically. To try to compensate for this difficulty, and to avoid side-effects, we used neuronavigation, i.e., we used MRI to guide the position of the TMS coil, and we used robotic positioning to enhance the precision of coil placement.

### 1.3 Rationale for the task used to investigate adjustments to sub-threshold trajectory manipulations

We used a new illusion discovered by Jovanovic et al. (2023). The task involved two squares moving towards each other and colliding, or stopping their trajectory only 1 pixel before collision. This task was chosen because former work suggested that thanks to the regularity of the trajectory, an illusion of gap emerges between the colliding squares (Jovanovic et al., 2023). The task thus allows to establish a relationship between the prediction, and the prediction error in case of a trajectory irregularity, and its consequence on a subjective level.

More concretely, the task allowed us to explore how a perturbation of the trajectory regularity affects the trajectory prediction and hence the illusion. When the perturbation is sub-threshold and undetectable consciously, it should be possible to ignore it. If not, the prediction should be affected. Since adjusting the prediction should take time, we introduced a trajectory perturbation less than 70 ms before the collision, i.e., a time too short to allow for a prediction adjustment. We thus expected to see the result of a lack of prediction rather than an adjusted prediction. Preliminary experiments confirmed an impact on the illusion rate when a trajectory acceleration occurred around 30 ms before the end of the trajectory (when the perturbation occurred earlier, the trajectory was regular again and there was no impact). If replicated, it would be an argument for the link between the millisecond-level prediction and the illusion, and would illustrate the perceptual consequences of prediction errors. Given the role of the cerebellum in the processing of prediction errors at the level of milliseconds, and in the processing of trajectories, we further tested our hypothesis with transcranial magnetic stimulation. We targeted the Crus I/II regions due to their role in trajectory prediction and perception, and their link with the fronto-parietal cortex (Guell and Schmahmann, 2020; King et al., 2019).

We measured electrophysiological (EEG) in addition to behavioral responses. Several EEG signals have been put in relation with the processing of prediction errors, like e.g., the N1 or MMN evoked potentials (Lieder et al., 2013; Pinheiro et al., 2019). Given the subconscious character of our manipulations, a MMN was not expected (but see (Liaukovich et al., 2022)). However, other signals may be observed. An update of predictions should follow a subliminal prediction error, and late signals have been described up to 1 s after a prediction error, like a decrease in beta oscillation, a feedback related negativity, or a late positive potential (Burnside et al., 2019; Chao et al., 2018; Hajcak and Foti, 2020). Given the new approach, it was difficult to predict what type of EEG results would be observed exactly, but EEG changes proved to be especially sensitive to the effect of TMS.

To summarize our objective and hypotheses, the present study documents the impact of sub-threshold, millisecond-level perturbations on conscious perception, by collecting a behavioral measure, EEG correlates, and by stimulating the cerebellum by means of TMS. The processing of trajectory prediction errors at the level of milliseconds was expected to impact the illusion rate and EEG signals, independent of TMS. Finally, we used TMS on the cerebellum to verify if the impact of the trajectory prediction errors can be modulated.

## 2 Methods

### 2.1 Participants

We recruited 24 healthy young adults (12 females, mean age ± SD: 24 ± 5 years) that were mainly students of the University of Strasbourg. The sample size was based on a previous study that evaluated the perception of the illusion (Jovanovic et al., 2023), where 16 participants were measured. Given the uncertainty of the TMS effect on the task, and the exploratory nature of the EEG analysis, we increased the sample size by 50%. Due to a lack of reliability in their data, we had to exclude 5 participants from the behavioural analyses, and 3 others from the EEG analyses (details are given down below). Given these limitations, we analyzed the results with Bayesian statistics. Using minimally informative priors, posterior distributions and credible intervals are driven by the data of our ∼20 participants, thus providing a transparent representation of the evidence and the respective uncertainty.

After having received detailed explanations about the experiment, participants gave their informed written consent and the study was conducted in accordance with the ethical agreements of Helsinki (World Medical Association, 2013) and approved by the ethical committee CPP IV Est (N° IDRCB: 2016-A00106-45).

### 2.2 Experimental procedure

**Fig. 1:**
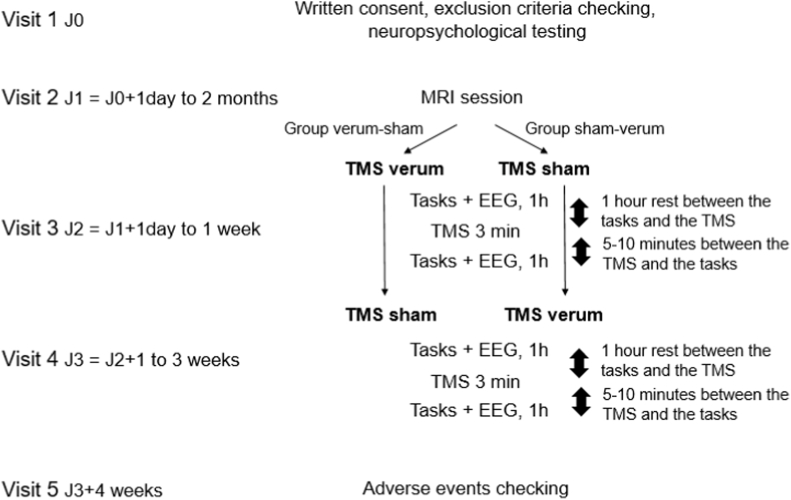
Experimental Procedure – Participants were invited to the lab 4 times: In visit 1, we explained the procedure, obtained the informed written consent, checked for exclusion criteria and performed neuropsychological tests. In visit 2, we measured participants in the MRI scanner, mainly to inform the TMS robot in the later intervention. We randomly assigned a group to each participant (counterbalanced between them), where in group verum-sham, participants received verum TMS on visit 3 and sham TMS on visit 4. In group sham-verum, participants received sham TMS on visit 3 and verum TMS on visit 4. Before and after the interventions, we measured behavioral and EEG responses. 4 weeks after concluding the experiments, we called participants to ensure that they did not experience any negative aftereffects.

Participants came to the lab 4 times (see Fig. 1). Once written consent had been obtained, we checked for exclusion criteria: (history of) neurological or psychiatric diseases, drug abuse, usage of psycho-active substances, take-in of benzodiazepines, cannabis consummation or other hallucinogen substances, decreased visual acuity (Bach, 1996), as well as contraindications for MRI and TMS. Participants were required to undergo both an MRI scan and TMS stimulation, and any contraindication to either technique was considered an exclusion criterion. This included the presence of permanent ferromagnetic body, pacemaker, prosthesis, vascular clip or stent, even if they were fMRI-compatible (due to the risk of heat or dysfunction induced by TMS), claustrophobia (for MRI), as well as pregnancy or breast-feeding. In the same meeting, we additionally ensured right-handedness via a handedness test (Oldfield, 1971) and measured neuropsychological abilities via the French National Adult Reading Test (Mackinnon and Mulligan, 2005), the CPT-AX (Cohen et al., 1999), a neurological test for soft signs in Schizophrenia (Krebs et al., 2000), which were within norms for all participants.

In the second meeting, participants were invited to a session in the MRI machine (Siemens Magnetom Verio 3T with a 32 channels head coil). We obtained anatomical data in order to later inform the neuronavigational system of the TMS robot. During this MRI session we also obtained functional MRI of the collision task and of another temporal prediction task (data not shown here).

In the third and fourth session, we measured behavioral and electrophysiological (Biosemi EEG system with 64 channels) data before and after transcranial magnetic stimulation (TMS) intervention. It should be noted, for the sake of reproducibility, that participants additionally performed a short version of the illusion task immediately before and after the intervention. We wished to explore the impact of the TMS on the illusion itself by exploring the rate of illusion in a condition in which this rate is low at baseline, i.e., when squares are drawn with outline contours (Jovanovic et al., 2023). We did not observe any change in the illusion rate. Moreover, there was no trajectory manipulation and the task unlikely changed the way trajectory perturbations were processed afterwards (Silvanto et al., 2007). The intervention was either the verum transcranial magnetic stimulation (TMS) or sham TMS (see more details below). Verum and sham TMS were performed on separate meetings and their order was counterbalanced between participants. We ensured a minimum of 6 days between the two meetings in order to prevent spill-over effects of the verum TMS on the sham TMS. The experimental part (before and after intervention) took place in the psychiatric hospital of the University of Strasbourg, while the intervention was performed at the CEMNIS, Strasbourg. The two locations are 200m apart from each other and the experimenters walked with the participant from one location to the other. Several EEG electrodes had to be unplugged in order to enable the TMS intervention, but the rest of the EEG setup remained on the participants’ head during the whole meeting.

As a final step, we called participants 4 weeks after completing the experiment and none of the participants showed any sign of (negative) after effects.

### 2.3 TMS

*Equipment:* rTMS was performed using a MagPro X100® with MagOption® (MagVenture, Farum, Denmark) and a 65-mm figure-of-eight coil (Cool-B65 A/P CO®, MagVenture, Farum, Denmark), which was water-cooled and equipped with navigation trackers and a pressure sensor to ensure compatibility with a robotic device for coil positioning (Axilum Robotics, Strasbourg, France). Both the stimulator and the robotic coil positioning device were controlled by a neuronavigation system (Localite GmbH, Sankt Augustin, Germany). Two stimulation targets were manually defined on an MP-RAGE 3D-T1 MRI scan (voxel size: 0.8 x 0.8 × 0.8 mm) based on anatomical features to target the Crus I and Crus II areas. The coil was oriented at 90° to the axis of the opposing sulci. The use of robotic assistance allowed for a neuronavigation error of less than 5 mm (Dormegny-Jeanjean et al., 2022).

*Verum TMS and sham TMS:* The transcranial magnet stimulation was a single intermittent theta-burst stimulation (iTBS) with 10 bursts of 3 biphasic pulses at 50 Hz, repeated at 5Hz and performed 20 times with 8s burst intervals (Huang et al., 2005). The total duration of the stimulation was 3min 22 s. Stimulations were executed via the Axilum Robotics TMS robot with the neuronavigation system Localite that was informed by the individual anatomical data obtained during meeting 2 (at least 7 days before meeting 3). We used MagVenture as the stimulator and a figure of 8 coil.

In order to account for individual scalp properties, we selected two possible targets, while aiming to stimulate the most lateral and more anterior part of the border (target 1). If this was not possible, a location more lateral and closer to intermediate region was stimulated (target 2). Target 1 was stimulated in 19 participants, while target 2 was stimulated in 5 participants. Given the small number of participants for which the target was more lateral, the following results are averaged across both targets. We always stimulated the right side because prior studies showed larger effects on sub-second perception duration or sensorimotor integration in the case of a stimulation on the right than on the left cerebellum hemisphere (Bijsterbosch et al., 2011).

For this study, we developed a procedure to estimate a stimulation threshold specifically adapted for the cerebellum. Our initial concern was that extrapolating stimulation intensity from the resting motor threshold (RMT) does not allow direct control over whether this intensity is sufficient to stimulate the cerebellum, given potential differences in excitability properties and coil-to-target distances (Hardwick et al., 2014). To empirically confirm that the stimulation-induced electric field could reach the cerebellar cortex and induce depolarization, we needed a physiological outcome, which, in the case of the cerebellum, should be negative (i.e., a disturbance of another process). Our reasoning was that if the electric field is sufficiently intense to reach the cerebellum, it should influence the parallel fibers, which are projected from the granular cells located in the intermediate lobe but extend into the lateral lobe. Such a stimulation should disrupt a continuous motor task, such as finger tapping, which can be assessed by an external observer – and used to determine the minimal stimulation threshold. The stimulation threshold was thus defined as the observation of a disruption of a continuous motor process (i.e., dyssynchronicity during a finger tapping task) induced by a single theta burst. Bursts were applied with incremental intensity (+2% of the stimulator's maximal output) to the most medial target used for the stimulation, with the same coil positioning as planned for the stimulation, until this disruption was observed by the operator.

For the iTBS intervention, the stimulation intensity was 100% of this cerebellar threshold. According to these parameters, the intensity used for our group should have been 59.3 ± 11.5% (mean ± SD) of the stimulator's maximum output. However, six participants experienced tolerance issues (muscle pain) during the verum stimulation and they required a reduction in intensity (10.3 ± 10.1%), resulting in an actual intensity of 56.8 ± 10.7% of the stimulator's maximum output.

In the sham TMS session, the coil was reversed such that the two currents cancel each other out. The employees at the TMS platform performed this intervention and were necessarily aware of the type of intervention, but the experimenters were not and they were also not present in the room during the intervention. It is to be noted that no participant had any nausea or muscle contracture following the TMS targeting the cerebellum, thus decreasing the chance that they discriminated between sham and verum TMS. Participants were not informed whether they received the verum or the sham TMS intervention. We could, however, not prevent that participants might have felt a difference due to (neck) muscle activation.

### 2.4 Equipment and apparatus

Participants were seated in a dimly lit and quiet room. A chinrest ensured the distance between head and screen of 67 cm. The stimuli and the procedure were programmed using MATLAB (version 2009b) and psychtoolbox on a HP Compaq 8100 Elite computer and presented on a CRT Sony CPD-520GST Trinitron monitor with a refresh rate of 60 Hz and a resolution of 1280x1024 pixels. Manual responses were recorded using the keys “f” and “j” on a standard keyboard.

### 2.5 Stimuli and experimental design

**Fig. 2:**
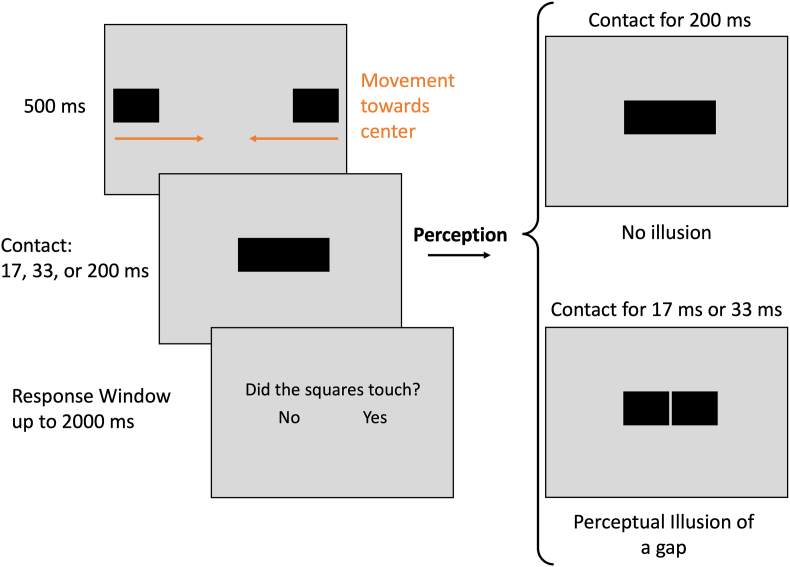
Two squares appear on the screen. They move towards the center until the inner edges of the squares touch for variable amounts of time (17, 33, or 200ms contact duration). The 200ms long contact duration was clearly visible and was used to ascertain that the participant followed instructions. With short contact durations (17 and 33ms), the touch of the squares is not necessarily perceived, which results in the perception of an illusory gap. Participants were asked to report whether they perceived the squares touching or not in a response time window following the contact.

We presented the recently developed collision task (Jovanovic et al., 2023) as illustrated in Fig. 2. In this visual illusion two black squares (1x1°VA, 0.07 cd/m^2^) were presented on a light gray background (8.9 cd/m^2^). They appeared 1° visual angle left and right of the middle of the screen. In the following 500 ms, the squares moved towards each other until their inner edges touched at the middle of the screen. The squares would then stay on the screen and disappear 17, 33, or 200 ms after their contact. After a 250ms waiting period, a response time window started in which participants were instructed to press one key in case the squares touched and another key in case the squares did not touch. The response time window was adapted to the contact duration, i.e., 1733 ms response window with 17 ms contact duration, 1717 ms response window with 33 ms contact duration, and 1550 ms response time window with 200 ms (no) contact duration. Afterwards, a fixed inter-trial interval (ITI) of 500ms was presented. The total duration of one trial was always 3000ms.

The illusion does not result from the brevity of the contact, since the contact is visible in several conditions even for 17 ms contacts, e.g., when squares are empty rather than filled in. Several control experiments led to the interpretation that the illusion originates from the violation of the low-level edge contrast predictions (Jovanovic et al., 2023). In detail, two black squares move towards each other on a gray background. The high-level, cognitive prediction would be that the squares touch in the middle of the screen, which is not what participants perceive in case of very short contact durations. It is rather the low-level prediction that seems to evoke the illusory gap through the regularity of the continuously moving edge contrast shift along the trajectory. According to this low-level prediction for position x+1, the inner edge of the left square should evoke a prediction of black on the left and gray on the right side of the edge, whereas for the right square it is gray on the left and black on the right. Even though the two squares touch, gray is predicted at the center of the screen. This illusory gap is what participants perceive, suggesting that the low-level predictions lead to this subjective perception.

Due to the previous study on the illusion (Jovanovic et al., 2023), we expected an illusion perception rate of roughly 60% in the 33ms trials (see supplementary material S2). We privileged 33ms trials (68% of all 300 trials), because it allowed to reliably measure both an increase and a decrease in illusion perception rate, i.e., any modulation of the illusion rate. The other 32% of the trials were evenly split between a contact duration of 200ms and a contact duration of 17ms. The 200ms trials served as control conditions, because with this long contact duration no illusion is perceived. In order to check that participants understood both key-meaning associations, we presented half of the 200ms trials with the squares touching in the middle of the screen, which is easily detectable as a contact. The other half of the 200ms trials was presented with the squares stopping their trajectory one frame, i.e., 1 pixel, before they would touch, which is easily detectable as a gap. The 17ms trials were presented because previously, an illusion perception rate of roughly 90% was measured with the 17ms trials. Those trials thus served as a control to ensure that participants understood the task in a condition in which the illusion was perceived with a high probability (as compared to the 200ms trials).

In the trials in which the trajectory was standard without perturbation, the squares moved 30 pixels within 500ms, resulting in 1 pixel per frame. Trajectory manipulation was introduced in some trials to disturb low-level predictions. In case of a perturbation, the squares moved 2 instead of 1 pixel within 1 frame. To avoid ending the trajectory on this 2-pixels-jump, the square moved one pixel more, meaning that the perturbation occurred 2 frames (33ms) before the contact. The squares then stayed on the screen for 17, 33 or 200 ms, and then disappeared. In most of the cases, this perturbation was not consciously perceived by the participants, as checked in the control condition presented in the supplementary material S1 (Fig. S1).

In the first block, we showed 100 trials that only contained non-perturbed trials (68 33ms-trials, 16 17ms-trials, 8 200ms-with contact, 8 200ms-without contact). In the second and third block, we presented 100 trials each. In each of these blocks, half of the trials containing non-perturbed and the other half of the trials containing perturbed trials.

The sequence of trials was determined using OptSeq2 (Dale, 1999) (10000 randomizations, optimal result taken for all participants; software available at https://surfer.nmr.mgh.harvard.edu/optseq/).

### 2.6 General principles of the Bayesian analysis

To quantify possible effects, we employed univariate Bayesian analyses using the RJAGS package (Plummer, 2003). Given that the behavioural data were naturally bounded and the ERP data exhibited non-symmetric distributions, assessed via visual inspection of histograms, we used a Bayesian Beta Regression approach, which is the method to use in case of linear and non-normally distributed data. Since the beta distribution is defined as an open interval ]0,1[, raw data were min-max-normalized and boundary values were avoided using the Smithson and Verkuilen (2006) transformation.

We used the logit link function to model the respective factors, as well as their interactions. All analyses were conducted using minimally informative priors. We used normally distributed priors for all main effects and intercept with mean 0 and precision 0.01. Two-way and three-way interaction terms were assigned broader normal priors with mean 0 and precision 0.001. To account for individual variability, participant-specific random intercepts were modeled as normally distributed with mean 0 and precision τ, where τ was assigned a Gamma prior with shape 0.1 and rate 0.1. The precision parameter of the beta regression was kept constant across factors and participants and defined as a Gamma prior with shape 0.01 and rate 0.01. Posterior distributions were obtained via the Gibbs sampling algorithm with 3 chains and 105,000 iterations, of which the first 5000 were discarded as burn-in. For each analysis and each factor, the first level was treated as a reference and fixed at zero on the logit scale. Differences between levels are reported as Odds Ratios (OR), calculated by exponentiating the estimated coefficients.

The probability for a difference of the respective comparison is denoted by e.g., *Pr*(verum TMS > sham TMS), i.e., that the performance is better with the verum TMS than with the sham TMS. For interactions, the probabilities are written as follows: *Pr*(OR > 1) with OR representing the exponentiated interaction coefficient. In case of meaningful interaction effects, we calculated pairwise posterior comparisons to isolate the origin of the effect. We define meaningful effects with values that are *Pr* > 0.975 or *Pr* < 0.025 since both are equivalent, as *Pr* (A > B) = 1 − *Pr* (A < B). In supplementary material S4 we provide convergence diagnostics for the conducted analyses. All Bayesian analyses were conducted using the R packages jags (Plummer, 2003) and brms (Bürkner, 2021).

### 2.7 Behavioral analysis

We calculated the illusion perception rate, i.e., the percentage of reports that the squares did not touch (which equals to an illusory gap). Two participants had less than 75% correct responses in either of the 200ms control conditions and were thus excluded from further (behavioural and EEG) analyses. Further, three participants had to be excluded due to abnormal perception in one of the trial types, i.e., one participant had 20% illusion perception rate instead of roughly 90% in the other trial types, and another 2 participants showed a floor effect of the illusion (less than 20% illusion perception rate) in the non-perturbed trials of the mixed blocks. We thus conducted the analyses on a total of 19 participants. The possibility to respond was given 250ms after the offset of the squares and thus we did not have to filter too short reaction time values.

For the behavioral analyses, we analyzed only the trials with 33ms contact duration at trial N. The experimental design included different types of trials (non-perturbed in two different blocks and perturbed trials) and several time points of measurement, i.e., before and after either verum or sham TMS interventions. Further, our design enabled a within comparison of the factors *Intervention* (verum or sham TMS) and *Time Point* (before or after the intervention) effects. As a result, half of the participants received the verum TMS in their first intervention meeting and the other half received sham TMS in their first intervention meeting. To investigate a possible influence of order of the sessions on the results, we additionally tested for the factor *Session Order* (Session 1 verum TMS, i.e., verum-sham, or Session 1 sham TMS, i.e., sham-verum). To facilitate the readers understanding of our analyses, we describe the details in the respective results part.

### 2.8 Electrophysiological analysis

*EEG recordings and pre-processing:* We measured the EEG with the ActiveTwo 64 channel BioSemi system with active silver/silver chloride electrodes. EEG data were digitized with a sampling rate of 2048 Hz. The data was offline down sampled to 512 Hz and digitally filtered with a low-pass at 25Hz and re-referenced to the common average. Data analysis was executed in Python using mne (Gramfort et al., 2013; Larson et al., 2024).

We detected bad electrodes by manually selecting those electrodes that showed an abnormal power spectral density. Bad electrodes are reported in the supplementary material, but did not affect the analyses (supplementary material S3). Blinks were detected using the mne ICA implementation. To do so, we filtered the data from 1 to 30 Hz, broke it down into epochs of 1s, and identified components as representing blinks when their correlation with electrodes Fp1 and Fp2 were equal or higher than a correlation coefficient of 0.8. In 14 out of 96 cases (24 participants, 4 EEG sessions each, i.e., before and after the two types of intervention) we had to manually correct the identified blink components. After removing those blink artefacts from the individual EEG data, we excluded trials from analysis when reaching an artefact threshold of ±150 μV. Amplitudes were measured relative to baseline, which was defined from 100ms before until the onset of the square's trajectory. Trials were calculated until 2900ms after the trajectory, i.e., until the end of the ITI. EEG data was sorted the same as in the behavioral analyses. We set a minimum of 20 trials per condition, participant, and trial type and averaged across illusion and no illusion trials. Due to problems during data acquisition, we had to exclude three out of the originally 24 participants from the EEG analyses. Given we excluded two participants because they did not comply to the task instructions (see the behavioral analysis) EEG analyses were conducted on 19 participants (albeit not exactly the same as for the behavioral analyses).

*ERP analyses:* The data were separately analyzed and averaged for each participant and for each EEG electrode using the onset of the trajectory as a time reference and then averaged across participants to obtain the grand mean data.

This study is somewhat explorative, since we had never measured EEG when presenting this visual illusion. We visually inspected the whole range of grand mean ERP data and found several temporal and spatial regions of interest. We selected the spatial and temporal region of interest that yielded the most meaningful results in terms of intervention influence, i.e., a difference between pre vs. post compared between verum TMS and sham TMS. This exploratory analysis revealed a differential effect of intervention at electrode CP4 in the time range from 0.15s until 2s after the contact of the squares, i.e., the whole response time window. We calculated the mean amplitude at electrode CP4 across the whole response time window (0.15s until 2s after stimulus onset) for each participant and condition (before verum TMS, after verum TMS, before sham TMS, after sham TMS) separately. This resulted in four amplitude values per participant, which were used to calculate the Bayesian statistics.

We conducted the same statistical comparisons as described in the behavioral analyses and the results section for these ERP amplitudes at CP4. Table 1 summarizes the analyses and the respective dependent variables.

**Table 1:** list of dependent variables.

Section	Analysis	Dependent variable
Results before TMS	Behavioural: *Trial type* x *Session* x *Session order*	Illusion rate
	EEG: *Trial type* x *Session* x *Session order*	ERP amplitude
Impact of the TMS	Non-perturbed trials (first block): behavioural: *Intervention* x *Time point* x *Session order*	Illusion rate
	Non-perturbed trials (first block): EEG: *Intervention* x *Time point* x *Session order*	ERP amplitude
	Non-perturbed trials (mixed block): behavioural: *Intervention* x *Time point* x *Session order*	Illusion rate
	Non-perturbed trials (mixed block): EEG: *Intervention* x *Time point* x *Session order*	ERP amplitude
	Perturbed trials: behavioural: *Intervention* x *Time point* x *Session order*	Illusion rate
	Perturbed trials: EEG: *Intervention* x *Time point* x *Session order*	ERP amplitude

### 2.9 Correlation of behavioral and ERP results

We calculated the correlations between behavioral and ERP results using Spearman rank correlations in R. This was done separately for the different conditions (non-perturbed trials in the first block, in the second/third block, and perturbed trials in the second/third block), types (verum TMS and sham TMS), and time points (pre vs. post) of interventions. Participants excluded from the behavioral analyses were not the same as those excluded in the EEG analyses. Reliable data in both measures was found in 16 participants, which were then used for calculating the correlations. We corrected for multiple testing using the Bonferroni method.

## 3 Results

### 3.1 Results before TMS

#### 3.1.1 Behavioural data before intervention

**Fig. 3:**
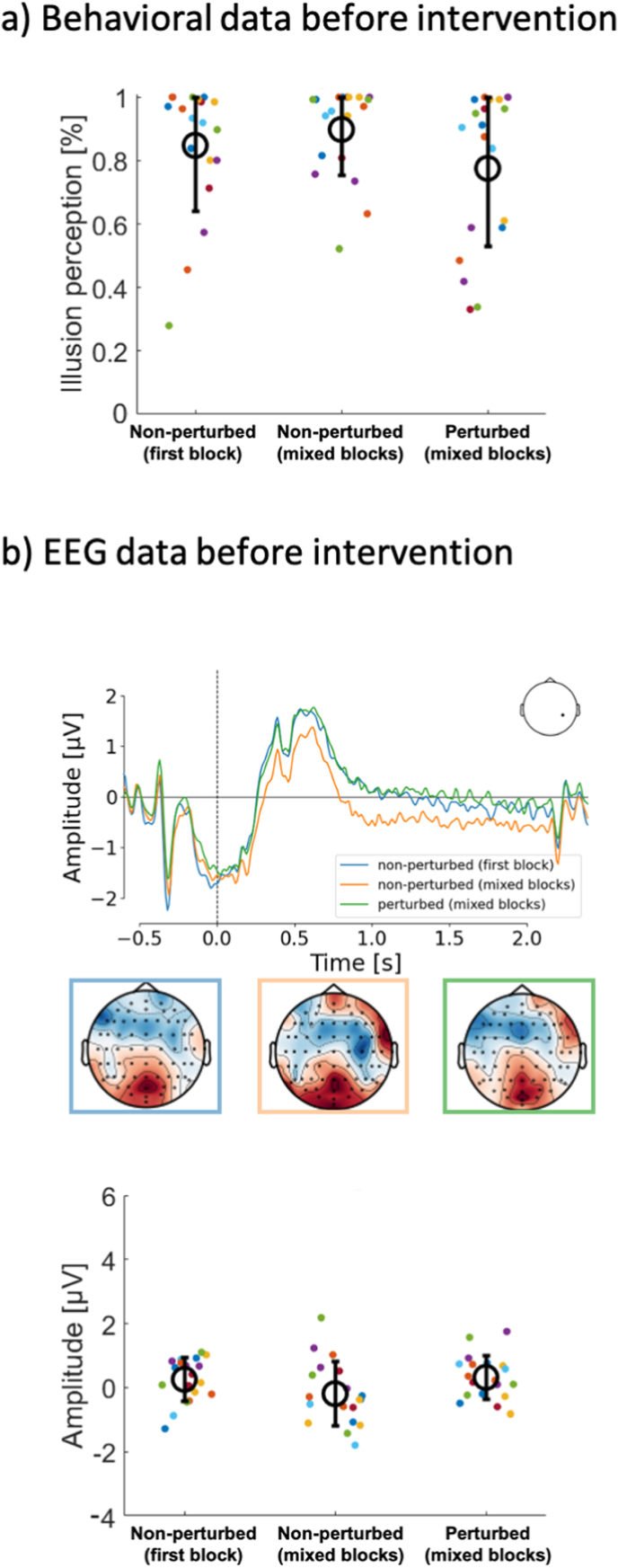
Data from the averaged morning sessions in response to the three trial types. In a) behavioral results are depicted, showing higher illusion perception rates for non-perturbed trials than for perturbed trials. In b) the grand mean ERP traces for electrode CP4 are depicted on the top, showing higher EEG amplitudes for perturbed (green line) than for non-perturbed trials (blue and orange lines). The topographical distributions observed 1 s after stimulus onset are illustrated for the different conditions below the ERP traces. On the bottom the respective individual EEG averaged amplitudes are depicted. In subgraphs depicting individual data, small colored circles represent individual participants and are independent of the color coding for the different conditions. Large empty black circles represent the mean and the error bars represent the standard deviation. Related scatter plots can be found in supplementary material S5.

Our first aim was to verify whether the trajectory perturbation impacted the illusion, irrespective of the TMS interventions (as illustrated Fig. 3a). To do so, we considered only data from the morning sessions. We investigated behavioral and EEG differences between trial types: 1) non-perturbed trials of the first block, in which solely non-perturbed trials were presented, 2) non-perturbed trials of the second and third block, i.e., those blocks in which non-perturbed and perturbed trials were mixed, and 3) perturbed trials of the second and third block (factor *Trial Type*). We analyzed the effects’ stability over time by comparing results from the morning of session 1 with that from the morning of session 2 (factor *Session*). However, there was either a verum or a sham TMS session in between the morning sessions, that might have influenced the data. We thus additionally tested for a possible difference between the two groups (factor *Session Order*), i.e., group receiving verum TMS on session 1 (group verum-sham) or on session 2 (group sham-verum).

We found that the illusion perception rate was clearly higher for the non-perturbed trials than for the perturbed trials. This was true for the illusion rate for perturbed vs. non-perturbed trials in the mixed block [N = 19; OR = 0.38, CI95%: 0.18-0.68, *Pr*(perturbed (mixed blocks) > non-perturbed (mixed blocks) = 0.001))]. This held true for perturbed trials in the mixed block vs. non-perturbed trials in the first block (with only non-perturbed trials) [N = 19; OR = 0.46, CI95%: 0.22-0.82, *Pr*(perturbed (mixed blocks) > non-perturbed (block 1)) = 0.006]. There was no difference between non-perturbed trials of the different blocks (N = 19; *Pr*(non-perturbed (mixed blocks) > non-perturbed (block 1)) = 0.7), see Fig. 3 a), irrespective of *Session* or *Session Order*. We did not find a main effect of Session (N = 19; *Pr*(morning 2 > morning 1) = 0.54), and no meaningful main effect of Session order (N = 19; *Pr*(verum-sham > sham-verum) = 0.08). We did not find any meaningful interactions, indicating that results did not change from morning 1 to morning 2, neither for the different levels of *Trial Type* nor of *Session Order*.

#### 3.1.2 EEG data before intervention

In the EEG data, we also found changes in ERP amplitudes as a function of the trajectory perturbations (as illustrated in Fig. 3b). We found higher ERP amplitudes at electrode CP4 in the time range of 0.15s to 2s after the contact of the squares, for perturbed trials compared to non-perturbed trials of the mixed blocks [N = 19; OR = 5.5, CI95%: 2.89-9.59, *Pr*(perturbed (mixed blocks) > non-perturbed (mixed blocks) > 0.99)]. Similar results were found when comparing the ERP amplitude at CP4 for perturbed trials in the mixed block to non-perturbed trials of the first block (with only non-perturbed trials) [N = 19; OR = 2.6, CI95%: 1.36-4.6, *Pr*(perturbed (mixed blocks) > non-perturbed (first block)) = 0.99]. We additionally found smaller amplitudes for non-perturbed trials in the mixed blocks compared to the first block [N = 19; OR = 0.5, CI95%: 0.27-0.83, *Pr*(non-perturbed (mixed blocks) > non-perturbed (first block)) = 0.004], see Fig. 3 b).

We did not find a meaningful change over time (N = 19; *Pr*(morning 2 > morning 1) = 0.48), nor between groups (N = 19; *Pr*(verum-sham > sham-verum) = 0.78). However, we found a meaningful interaction between *Trial type* and *Session* (N = 19; OR = 0.27, CI95%: 0.11-0.57, *Pr *= 0.001), which resulted from a decrease in amplitude values from the first to the second morning for perturbed trials (N = 19; OR = 0.26, CI95%: 0.13-0.44, *Pr* < 0.001). In contrast, there was no evidence of a change in amplitudes for the non-perturbed trials (N = 19; *Pr* = 0.48 and *Pr* = 0.965, for non-perturbed trials of the first and the mixed block, respectively).

Other meaningful interactions suggested that the EEG signals changed over time in case of perturbed trials, depending on the Session Order (sham-verum vs. verum sham) as well as the session (1st or 2nd morning). There was an interaction between *Trial Type* and *Session Order* (N = 19; OR = 0.42, CI95%: 0.17-0.87, *Pr* = 0.01), together with a meaningful 3-way interaction (N = 19; OR = 4.9, CI95%: 1.3-12.76, *Pr* = 0.99). There were two modulations specific to perturbed trials. First in the group sham-verum, amplitudes decreased from the first to the second morning for perturbed trials (N = 9; OR = 0.26, CI95%: 0.13-0.44, *Pr* < 0.001), which was not the case in the non-perturbed trials (N = 9; *Pr* = 0.48 and *Pr* = 0.965, for non-perturbed trials of the fist and the mixed block, respectively). Second, on the second morning, amplitudes where higher for the group verum-sham (N = 10) than for the sham-verum (N = 9) for perturbed trials (OR = 7.2, CI95%: 1.14-24.3, *Pr* = 0.98). This was not the case in the non-perturbed trials (*Pr* = 0.78 and *Pr* = 0.47, for non-perturbed trials of the first and the mixed block, respectively).

There was additionally one main effect of group that concerned non-perturbed trials in the mixed block. EEG amplitudes were smaller in non-perturbed trials in the mixed block in the group running TMS in the sham-verum order (N = 9) rather than in the verum-sham order (N = 10) (OR = 2.19, CI95%: 1.1-3.99, *Pr* = 0.987). There was no such difference in the first block with only non-perturbed trials (*Pr* = 0.78).

The results above confirm an effect of the perturbation on both the illusion rate and the EEG signals, with a decrease of the illusion rate, and an increase of the ERP amplitude in CP4. We found meaningful effects suggesting different evolutions across time depending on *Trial types*, which could confound with effects of the TMS interventions. We thus separately investigated the influence of interventions for the three trial types: non-perturbed (first block), non-perturbed (mixed blocks), perturbed (mixed blocks). Further, the meaning of a TMS impact on perturbed and non-perturbed trials is different. If TMS changes the illusion itself, e.g., by affecting the processing of contrast prediction errors, then it should affect the results observed in non-perturbed trials. If TMS affects the processing of the trajectory prediction error, then it should affect the results observed in perturbed trials.

### 3.2 Impact of the TMS

We investigated the influence of the TMS interventions on the illusion perception rate and on EEG data by means of the factors *Intervention* (verum and sham TMS), *Time Point* (before and after intervention), and Session Order (verum-sham vs. sham-verum) separately for the different *Trial Types:* non-perturbed (first block), non-perturbed (mixed blocks), and perturbed (mixed blocks). As already emphasized, we analyzed non-perturbed and perturbed trials separately, and present behavioural and EEG results for each type of trials in turn.

#### 3.2.1 Non-perturbed trials

***Behavioral results:***

The data of the non-perturbed trials in block 1 (with only non-perturbed trials) showed only one marginal effect. The detailed results can be found in supplementary material S6.1. In a next step, we analyzed data of non-perturbed trials in the mixed blocks.

**Fig. 4:**
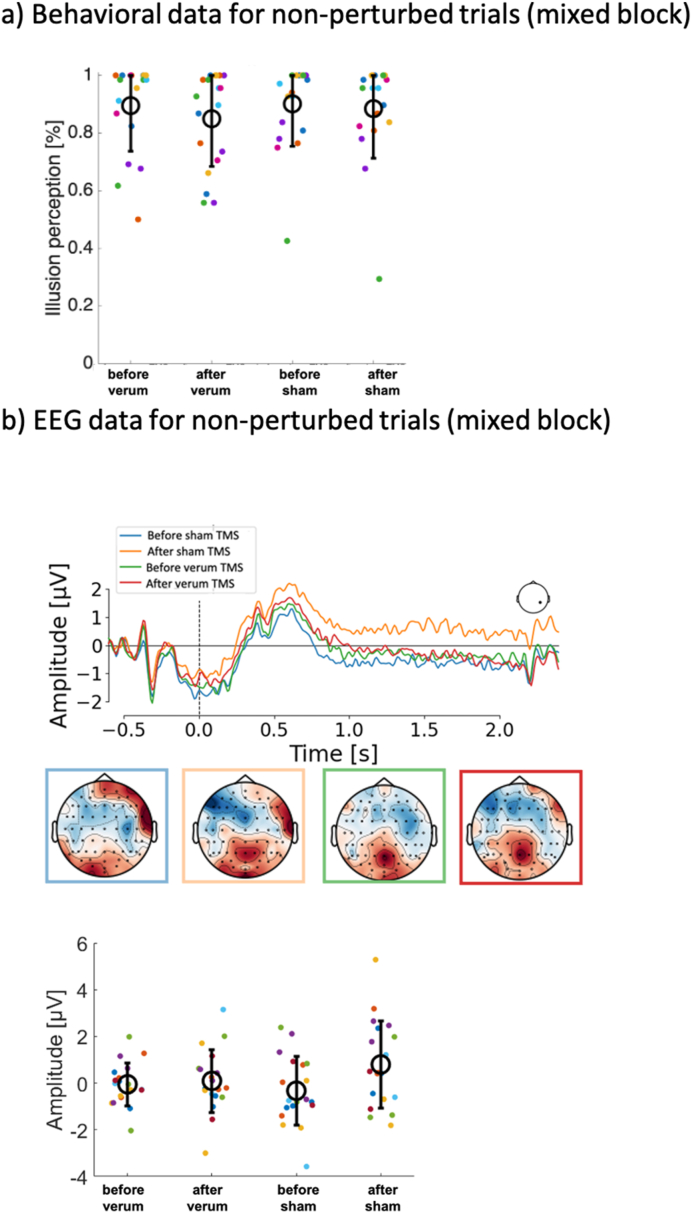
Non-perturbed trials of the mixed blocks – a) behavioral data, i.e., the illusion rate, is illustrated as a function of the Time point (before vs. after stimulation) and type of intervention (verum vs. sham) b) EEG data is illustrated with the grand mean ERP data on the top. The topographical distributions observed 1 s after stimulus onset are illustrated for the different conditions below the ERP traces. Individual data is depicted on the bottom. Like for behavioural data, EEG data is displayed as a function of the Time point (before vs. after stimulation) and type of intervention (verum vs. sham). In subgraphs depicting individual data, small colored circles represent individual participants and are independent of the color coding for the different conditions. Large empty black circles represent the mean and the error bars represent the standard deviation.

We found no effect of *Intervention* (N = 19; *Pr*(verum TMS > sham TMS) = 0.49), no effect of *Time Point* (N = 19; *Pr*(after > before) = 0.2), and no meaningful interaction (N = 19; *Pr* = 0.25). Further, *Session Order* did not show any meaningful effects (N = 19); *Pr*(verum-sham > sham-verum) = 0.11) nor interactions, see Fig. 4 a).

***EEG results:***

In the EEG data (illustrated Fig. 4b), we did not find a meaningful effect of *Intervention* (N = 19; *Pr*(verum TMS > sham TMS) = 0.83), but amplitudes increased from before to after TMS [N = 19; effect of *Time Point*: OR = 2.04, CI95%: 1.28-3.1, *Pr*(after > before) = 0.99] independently of the type of *Intervention*. We additionally found a meaningful interaction between *Intervention* and *Time Point* indicating an increase of amplitudes from before to after sham TMS (N = 19; OR = 2.04, CI95%: 1.28-3.1, *Pr* = 0.99), which was not the case for verum TMS (N = 19; *Pr* = 0.56), see Fig. 4 b). Regarding *Session Order*, we did not find a main effect (N = 19; *Pr*(verum-sham > sham-verum) = 0.94), and *Session Order* only interacted with *Time Point* (N = 19; OR = 0.45, CI95%: 0.17-0.98, *Pr* = 0.023), but not with *Intervention* (N = 19; *Pr* = 0.19), nor was there a meaningful three-way interaction (N = 19; *Pr* = 0.903).

In all there was no evidence of an effect of the intervention on behavioural responses to non-perturbed trials, and only a decrease in the amplitude of the EEG signal after a sham stimulation, and no change after a verum stimulation.

#### 3.2.2 Perturbed trials

***Behavioural results:***

**Fig. 5:**
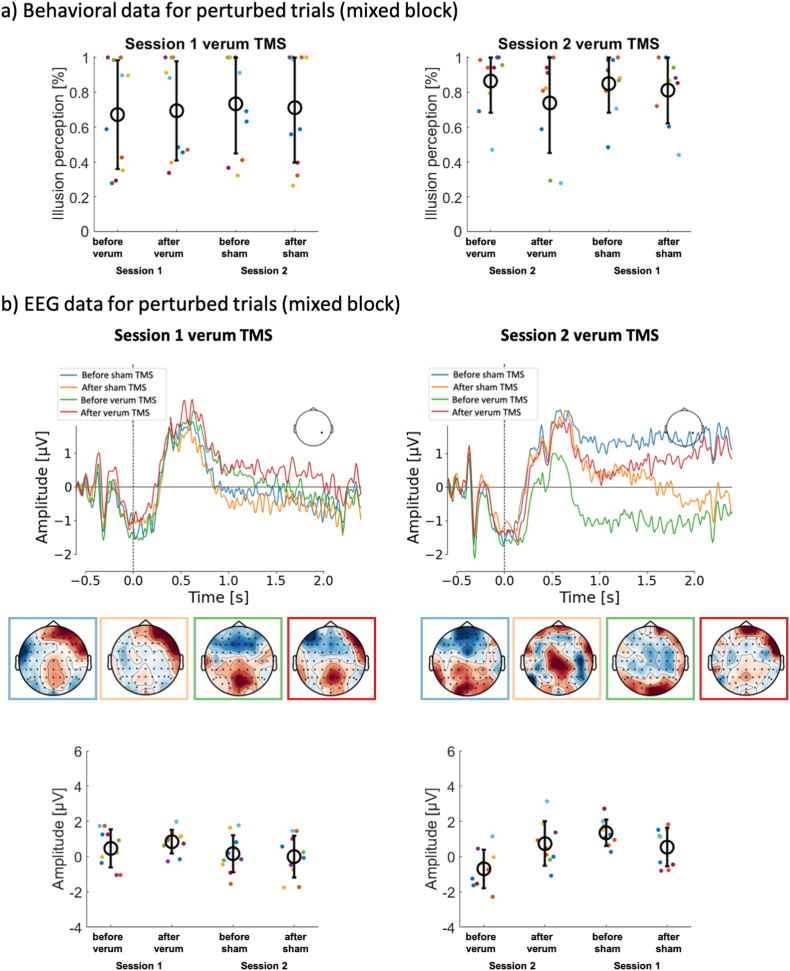
Perturbed trials of the mixed blocks –a) behavioral data, i.e., the illusion rate, is illustrated as a function of the Time point (before vs. after stimulation) and type of intervention (verum vs. sham). The illusion rate decreased from before to after intervention for the group receiving verum TMS at session 2 (right graph), whereas illusion rates stayed constant for the group receiving verum TMS at session 1 (left graph). b) EEG data is also illustrated as a function of the Time point (before vs. after stimulation) and type of intervention (verum vs. sham). We depicted grand mean ERP data on the top. The topographical distributions recorded 1s after stimulus onset are depicted below the ERP data, and the individual data is depicted on the bottom. For both groups, we found that amplitudes decreased from before to after sham TMS. For verum TMS, amplitudes slightly decreased from before to after in the group receiving verum TMS at session 1 (left graph), but they increased in the group receiving verum TMS at session 2 (right graph). Small colored circles represent individual participants and are independent of the color coding for the different conditions, large empty black circles represent the mean and the error bars the standard deviation.

We investigated perturbed trials (see Fig. 5a) and found that neither *Intervention* (N = 19; *Pr*(verum TMS > sham TMS) = 0.26), nor *Time Point* (N = 19; *Pr*(after > before) = 0.24), nor an interaction (N = 19; *Pr* = 0.28) had meaningful effects on the illusion rate. *Session Order* did not show a meaningful effect (N = 19; *Pr* = 0.33), nor meaningful interactions (N = 19; *Pr* between 0.06 and 0.95), see Fig. 5 a).

We did not find a meaningful three-way interaction in the behavioral results of the perturbed trials, but we did find such an interaction in the EEG, showing clear results for the group sham-verum (see the results in the next paragraph). In a very exploratory, additional, analysis, we quantified possible behavioral effects for the group sham-verum and found a decrease of illusion from before to after verum TMS intervention (N = 9; *Pr*(after verum TMS for sham-verum > before verum TMS for sham-verum) = 0.002), but no difference in behavior for the sham TMS intervention (N = 9; *Pr*(after sham TMS for sham-verum > before sham TMS for sham-verum) = 0.24).

***EEG results:***

Regarding EEG amplitudes, perturbed trials (see Fig. 5 b) showed several meaningful effects, which can be inspected in supplementary material S6.2, but here we want to highlight a clearly meaningful interaction between *Intervention* and *Time Point* (N = 19; OR = 3.3, CI95%: 1.5-6.4, *Pr* > 0.999), revealing an increase in amplitudes from before to after verum TMS (N = 19; OR = 2.25, CI95%: 1.3-3.65, *Pr*(after verum TMS > before verum TMS) > 0.999), but no such amplitude change for sham TMS (N = 19; *Pr*(after sham TMS > before sham TMS) = 0.04). Interestingly, we also found a meaningful three-way interaction between *Intervention*, *Time Point* and *Session Order* (N = 19; OR = 0.27, CI95%: 0.06-0.82, *Pr* = 0.012), showing that the increase from before to after verum TMS was found only when it was applied on the second session (N = 9; OR = 4.2, CI95%: 1.95-8.02, *Pr*(after verum TMS for sham-verum > before verum TMS for sham-verum) = 0.999), but not when it was applied on the first session (N = 10; *Pr*(after verum TMS for verum-sham > before verum TMS for verum-sham) = 0.38), see Fig. 5 b).

#### 3.2.3 Correlation between behavioral and electrophysiological results

We did not find any significant correlations between behavioral and electrophysiological results by means of Spearman rank correlation coefficients in any of the measures.

## 4 Discussion

The main result is that a small and undetectable perturbation of a linear motion trajectory impacts the conscious illusion rate as well as EEG signals. The impact of the perturbation on the illusion rate is stable from the first to the second morning session, while the EEG signals decrease from the first to the second morning session, and specifically so in case of perturbed trials. Despite the brevity of the stimulation session, TMS on the cerebellum modulates EEG responses to perturbed trials. These changes occur depending on the *Session Order* (verum-sham vs. sham-verum), and there is a behavioural impact of TMS only in an exploratory sub-analysis. These limitations necessarily restrain our understanding of how precisely the cerebellum is involved, but the results nonetheless suggest its involvement in case of a trajectory perturbation.

The main effect remains the impact of the trajectory perturbation on the illusion. Subliminal manipulations are known to affect behavioural responses and brain activation, like in priming studies (Pessiglione et al., 2007; Van den Bussche et al., 2009). Previous studies have also shown that sub-threshold prediction errors elicit EEG signals, often early ones (Rowe et al., 2020; Teixeira et al., 2020). It is usually believed that the processing of sub-threshold prediction errors can help adjusting learning (to perceive or to move) (Bavassi et al., 2013; Rose et al., 2005). To which amount such errors have an impact on consciousness is less clear (but see Foerster et al., 2021; Weibel et al., 2013). In itself the illusion used in the present study shows the conscious consequence of a sub-threshold prediction error (Jovanovic et al., 2023). As a reminder, we hypothesized that the regularity of the trajectory allows for the illusion, by making it possible to predict the future position of the two squares, together with the figure-ground contrast of their leading edge. When the two squares touch, the figure-ground contrast disappears, as the two squares are drawn with the same gray level (see Jovanovic et al., 2023; Jovanovic et al., 2023 for different, control, situations). The disappearance of the figure-ground contrast represents a prediction error, but this error is not consciously perceived. On the contrary, participants do not see the contact, and instead see a gap between the squares.

The fact that a perturbation of the trajectory reduces the illusion supports the hypothesis that the trajectory regularity plays a crucial role in the illusion. What is noteworthy in the present results is that the impact of the trajectory manipulation is almost immediate. When the square jumps by 2 instead of 1 pixel, it takes 17ms, and 17 ms for a last 1-pixel-move. The square then stops for 34 ms and disappears. There is thus 68 ms between the start of the jump and the disappearance of the square. Despite this short delay, the jump appears to facilitate the detection of a contact between the two squares. It is unlikely an attention effect. First, the jump is undetectable (see Supplementary Material S1) and 68 ms is too short to displace attention (VanRullen, 2018). It is also too short to allow for a top-down modulation effect (Supèr et al., 2001). Additionally, the jump means that the squares disappear 17 ms earlier than in the non-perturbed trials. This should have made it more difficult to see the contact between the two squares. It is thus all the more remarkable that the illusion decreases, meaning that the participants see the contact more frequently in case of a perturbed trajectory. Although counter-intuitive, the results are consistent with the idea that the trajectory perturbation disturbs the trajectory prediction itself. The 68 ms delay before squares’ disappearance does not allow to readjust the prediction online. The disturbance of the trajectory temporarily suspends prediction, which makes it more difficult to emit a prediction error and thus to evoke the illusion.

The decrease in the illusion rate was accompanied by an increase in a positive EEG signal. This signal looks like a late positive potential (LPP) (Hajcak and Foti, 2020), at least the type of LPP signal that is observed in case of a neutral stimulus, i.e., with a slowly decreasing amplitude. However, the late EEG signal is mainly described in response to emotional prediction error, i.e., threat (Botelho et al., 2023; Del Popolo Cristaldi et al., 2021), which does not seem to play a major role in our study. Some studies show a LPP independently from an emotional response (Hajcak et al., 2010; Hofmann et al., 2019; Jahshan et al., 2015; MacKay et al., 2024; Sun et al., 2017) and in paradigms with moving stimuli, MacKay et al. (MacKay et al., 2024). However, at this stage our interpretation of the positive signal in our study can only be speculative. For example the EEG signal might be related to the stimulus significance (Bradley, 2009, ; Hajcak and Foti, 2020), i.e., the significance of the change in trajectory. We might tentatively propose that in our study, the late EEG signal conveyed the importance given to the trajectory change. It might have been expected that such signals are associated with significant trial-to-trial changes in behavioural responses, which was not the case, see supplementary material S6.3. The randomization of the trials, which included both the randomization of trajectory perturbations, but also contact times, probably made this information less and less pertinent with time. This might explain the decrease in the EEG signal from the first to the second morning in case of a perturbation, but this hypothesis will have to be explored in further studies.

The impact of the sub-threshold irregularities is of interest for different pathologies. Abnormalities in the processing of asynchronies and delays in schizophrenia have been observed at the level of milliseconds (Foerster et al., 2021; Lalanne et al., 2012; Marques-Carneiro et al., 2021), and EEG data has similarly led to the hypothesis of abnormalities at this timescale (Lechner et al., 2025). An abnormal sensitivity to low-level prediction errors has also been proposed in autism (Van de Cruys et al., 2014). According to the HIPPEA (High Inflexible Precision of Prediction Errors in Autism) model (Van de Cruys et al., 2014), it might be related to a difficulty to modulate the processing of prediction errors, either by ignoring them or by increasing their significance. In both pathologies, the abnormal processing of sub-threshold signals would have an impact on conscious experiences, and especially abnormal perceptions (Giersch et al., 2016; Van de Cruys et al., 2014). Our results confirm that visual processing at the millisecond timescale indeed can affect conscious experiences. Unravelling the processing mechanisms at this timescale may help to understand the subjective experiences of patients.

TMS affected the EEG signals, and specifically so in case of a trajectory perturbation. As a matter of fact, it should be noted that in the absence of any perturbation, most effects of the TMS on the illusion rate or EEG signals were marginal and differed in the first block and the mixed blocks. We did not expect that TMS on the cerebellum would affect the illusion itself, given figure-ground contrast is processed elsewhere than in the cerebellum. In fact, the only clearly significant results of TMS were found on EEG signals in the mixed block, and appear to mirror the effects in case of a trajectory perturbation. In case of a perturbation, the amplitude of the late positive ERP signal significantly increased after the stimulation, at least in participants being stimulated with a verum TMS on session 2. When conducting a very exploratory analysis in this group (sham-verum), we found a decrease of illusion rate after the stimulation. In contrast, in case of non-perturbed trials in the mixed blocks, the LPP decreased after the sham TMS, but remained stable after the verum TMS. The association between an increase in amplitude of the late positive ERP signal and the drop in the illusion rate for perturbed trials match the results observed in session 1 in the morning. This parallelism suggests that the stimulation of the cerebellum led to increased responsiveness to the millisecond-level trajectory perturbation. Such an impact coheres with the literature suggesting a role of the cerebellum in the processing of prediction errors (Brooks and Cullen, 2013; Brooks et al., 2015), motion (Ivry and Diener, 1991; Thier et al., 1999), motion trajectory prediction (Baumann et al., 2015; Baumann and Mattingley, 2010; O'Reilly et al., 2008), and millisecond-level timing (Coull et al., 2011; Ivry and Spencer, 2004). The results are consistent with our interpretation that the illusion comes about as a result of trajectory prediction, and decreases due to the millisecond-level trajectory perturbation.

Despite the coherence of the results with the literature, and the use of one single and short-duration stimulation, which likely reduced its impact, the effect of *Session Order* should be discussed. As a matter of fact, no clear impact of the TMS is observed in those participants who had a verum TMS on the first session. A general group effect is not supported by the data since the group did not reveal meaningful effects in any of the behavioral analyses, except for the special case of perturbed trials described above. An explanation may thus be tentatively found in relation with the ERP signal. If it reflects the significance of the perturbation, TMS would result in an increase of the saliency of the perturbation. It may have reinstated a significance during session 2, that had worn off over time, as suggested by the decrease in this ERP amplitude from session 1 to session 2. However, at this stage this interpretation is at best speculative, and there are alternative possibilities, like an effect on motivation, fatigue or attention. The fact that the amplitude of the EEG evoked response, as well as the behavioural impact rather increased in case of trajectory perturbations does not cohere with those alternatives, but they still should be carefully examined. It might also be proposed that verum TMS increased alertness, but it is not clear why this should occur more in the second session than in the first session. In any case, the limitations of the present work regarding the TMS effects restrain our interpretation on its effects, and should be cared for in future studies. First, although participants were tested several times, the training before the first TMS session on the collision task was scarce. A lack of performance stabilization might explain the order effects. Second, the stimulation should be longer, and the blinding procedure might be replaced by a comparison between two different sites stimulations, or a comparison between a stimulation during the task vs. independent of the task. Our TMS threshold calculation was based on a cerebellum-related effect (sequential tapping), which is innovative and justified by the fact that we stimulate the cerebellum. However, it is unusual and will have to be tested more systematically. Also, we had to slightly change the cerebellum target in a few participants, with a more lateral stimulation, and the impact of this difference should be investigated. Finally, the use of a task with a longer period before the collision may help to explore EEG signals preceding the collision and not only late signals observed after the collision.

In conclusion, we provide evidence that the manipulation of a square's moving trajectory at the millisecond-level affects the processing of the trajectory within the 100 ms following the trajectory perturbation, very probably by interrupting the trajectory prediction. This effect occurs even as the manipulation remains sub-threshold. Despite the sub-threshold and non-conscious character of the manipulation, it affects conscious perception, as evidenced by the consequence on the rate of the illusion. The results thus confirm that the processing at the level of milliseconds is not restricted to automatic adjustments. The perturbation was not ignored, as might have been expected if trajectories are verified attentionally, with cycles of 100 ms (VanRullen, 2018). It remains to be explored if the fact that the perturbation occurred always at the same time, and at the end of the trajectory played a role in the effects, and to which extent the time resolution of trajectory checking mechanisms is always at the level of milliseconds. The manipulation further impacts EEG signals, in the form of a positive ERP signal. Also, the stimulation targeting the Crus I/II in the cerebellum affects behaviour slightly and more clearly the late ERP signal, at least in those participants who received verum TMS after having performed the task three times. Those results suggest the involvement of the cerebellum, but its precise role will have to be assessed more firmly in future studies.

## CRediT authorship contribution statement

**Ellen Joos:** Data curation, Formal analysis, Investigation, Methodology, Visualization, Writing – original draft. **Camille Scherer:** Investigation, Methodology. **Philippe Isope:** Conceptualization, Funding acquisition, Writing – review & editing. **Jack Foucher:** Conceptualization, Investigation, Methodology. **Anne Giersch:** Conceptualization, Funding acquisition, Methodology, Supervision, Validation, Writing – review & editing.

## Declaration of competing interest

The authors declare that they have no known competing financial interests or personal relationships that could have appeared to influence the work reported in this article.

## Data Availability

We share the link to the code and research data
