# Supplementary material for: Subthreshold violations of trajectory predictions are sensitive to TMS of cerebellum Crus I/II
Source: Neuroimage Rep. 2026 Jul 20;6(3):100382. doi: 10.1016/j.ynirp.2026.100382 (PMC13392610; doi:10.1016/j.ynirp.2026.100382)
Supplement: Multimedia component 1 [file mmc1.docx]

Supplementary Material for: Subthreshold violations of trajectory predictions are sensitive to TMS of Cerebellum Crus I/II

Supplementary material S1: Perturbation detection

Perturbations of the squares’ trajectory take place 2 frames before they contact each other in the middle of the screen. In a separate control experiment, we asked participants to answer two consecutive questions after each stimulus. They first indicated whether the squares touched or not and then indicated whether they perceived a perturbation or not. With this we aimed to test whether the perturbation reached a conscious level. We presented the same ratio of 17ms, 33ms, and 200ms trials as in the perturbation condition described in the main manuscript, but we only presented 100 trials overall, with half of the trials containing a perturbation and the other half not. In order to not bias participants towards consciously perceiving perturbations in the main results, we performed this control experiment at the end of the second measurement day, i.e., after finishing all the other experimental conditions.

As can be seen in Fig. S1, participants show similar rates of perturbation perception for trials containing perturbations vs. not (Wilcoxon signed rank test: *Z*=-1.14, *p*=0.26), suggesting that they did not reliably detect perturbations. These results suggest that the data reported in the main manuscript is not related to a conscious perception of the perturbation.

| 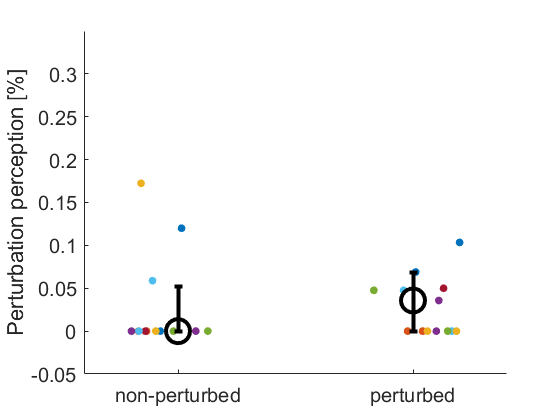 |
| --- |
| Fig. S1 The rate of perturbation perception in a control condition is displayed for 33ms trials. On the left the control trials with no perturbation show the baseline for perturbation perception in case of its absence. On the right, the results concern the trials including a perturbation. Small colored circles represent individual data, large black empty circles the median and the error bars the interquartile ranges. |

Supplementary Material S2: Trial type selection

In the collision task, two squares move towards each other until their inner edges touch in the middle of the screen. In a previous study (Jovanovic et al., 2023), it was found that in case of short (17ms and 33ms) contact durations this can result in an illusory perception of a gap between the two squares.

We did not have any a priori assumptions to decide whether the TMS intervention would result in an increase or in a decrease of the illusion perception rate in case of a trajectory manipulation. In a previous study (Jovanovic et al., 2023) (see also Fig. S2), the illusion perception rate for 17ms contact duration was 89.6% (SD: 3.9%) and for 33ms contact duration it was 67.1% (SD: 7.5%). We thus selected the 33ms contact duration trials to constitute the majority of trials in the currently conducted study, allowing us to reliably measure both a possible increase or a possible decrease of the illusion perception rate.

| 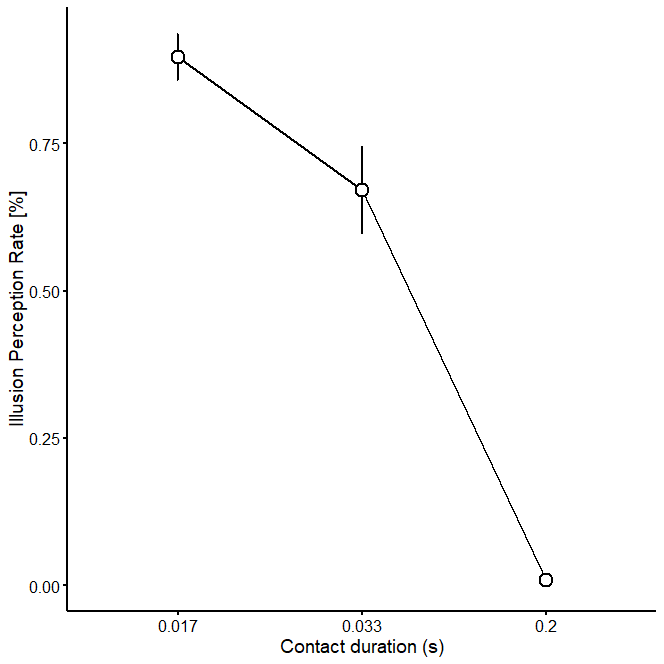 |
| --- |
| Fig. S2 Illusion in a previous study. The illusion perception rate is decreasing with increasing contact duration. Circles represent the mean value across 12 participants and error bars the standard deviation. |

Supplementary Material S3: bad electrodes

CP4 was never excluded/interpolated. The following is a list of the bad electrodes for each participant and each condition.

| **Participant** | **Condition** | **Bad Electrodes** |
| --- | --- | --- |
| 1 | before sham | Fp1 |
| 1 | after sham | None |
| 1 | before verum | None |
| 1 | after verum | None |
| 2 | before verum | None |
| 2 | after verum | None |
| 2 | before sham | Fp1 |
| 2 | after sham | T7, Fp1, Fp2, Fpz, AF7 |
| 3 | before verum | None |
| 3 | after verum | F4 |
| 3 | before sham | AF8, Fp2, F8, AF7, Fpz, Fp1, F6, AF4 |
| 3 | after sham | AF8 |
| 4 | before verum | None |
| 4 | after verum | None |
| 4 | before sham | None |
| 4 | after sham | None |
| 5 | before sham | AF7, AF8 |
| 5 | after sham | AF7, F7, Fp1, AF8 |
| 5 | before verum | AF7, Fp1, AF8 |
| 5 | after verum | None |
| 6 | before sham | None |
| 6 | after sham | None |
| 6 | before verum | None |
| 6 | after verum | None |
| 7 | before verum | Fp2, AF7, Fp1, T8, AF4, AF3, F7, FC3 |
| 7 | after verum | None |
| 7 | before sham | None |
| 7 | after sham | None |
| 8 | before sham | Iz |
| 8 | after sham | Iz, F2, F7 |
| 8 | before verum | None |
| 8 | after verum | None |
| 9 | before verum | None |
| 9 | after verum | None |
| 9 | before sham | None |
| 9 | after sham | None |
| 10 | before sham | None |
| 10 | after sham | F7, AF7 |
| 10 | before verum | T8, T7, FT8 |
| 10 | after verum | None |
| 11 | before verum | None |
| 11 | after verum | None |
| 11 | before sham | None |
| 11 | after sham | AF8 |
| 12 | before sham | AF8, F8 |
| 12 | after sham | AF8, AF7 |
| 12 | before verum | PO7, AF7, AF8, AF3, F7, Fpz, FT7, F4, Fp2, O2 |
| 12 | after verum | AF7, F7 |
| 13 | before sham | None |
| 13 | after sham | None |
| 13 | before verum | None |
| 13 | after verum | None |
| 14 | before verum | None |
| 14 | after verum | None |
| 14 | before sham | None |
| 14 | after sham | None |
| 15 | before sham | PO7, Iz, AF7 |
| 15 | after sham | AF7 |
| 15 | before verum | AF7, F7, Fp1, O1, FC6 |
| 15 | after verum | AF7, F7, Fp1 |
| 16 | before verum | Fp1, Fp2, AF8, AF7, Fpz |
| 16 | after verum | Fp1, Fp2, AF8, AF7, Fpz |
| 16 | before sham | None |
| 16 | after sham | TP7 |
| 17 | before sham | None |
| 17 | after sham | AF4, AF8, F4, C2, Fz |
| 17 | before verum | AF7, Fp1, Fpz |
| 17 | after verum | Fp2, AF8, Fpz, Fp1 |
| 18 | before verum | P3 |
| 18 | after verum | None |
| 18 | before sham | AF7, AF8, Fp2, Fpz, Fp1, AF4, F8 |
| 18 | after sham | AF8, Fp2, Fpz, AF7, Fp1, AF4, F8, F6, AFz, F4, AF3 |
| 19 | before verum | AF8 |
| 19 | after verum | None |
| 19 | before sham | None |
| 19 | after sham | None |
| Table S1 – bad electrodes | | |

Supplementary Material S4: Convergence diagnostics

| Analysis | | Traceplot |
| --- | --- | --- |
| Results before TMS | Behavioral data before intervention | 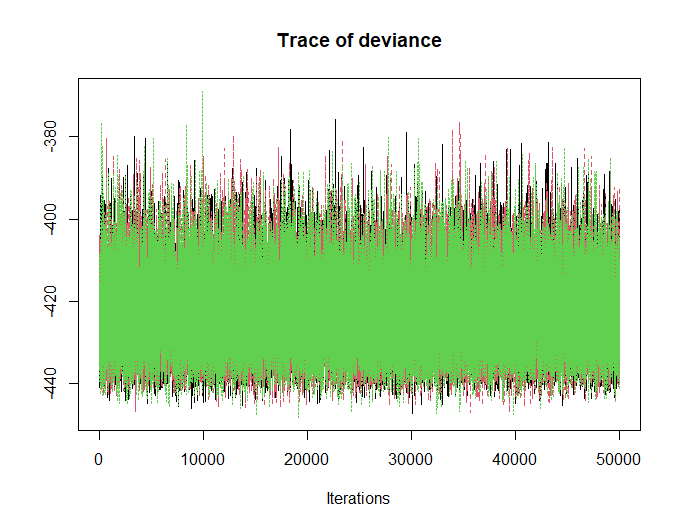 |
|  | EEG data before intervention | 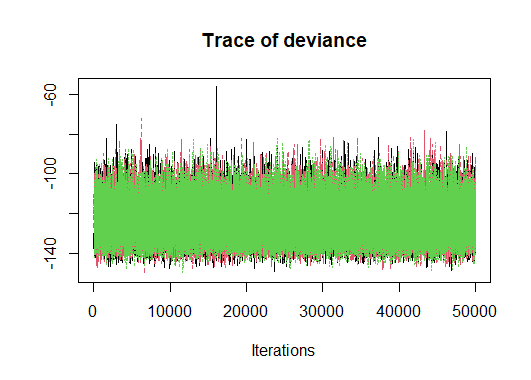 |
| Impact of the TMS | Non-perturbed trials (block 1): behavioral results | 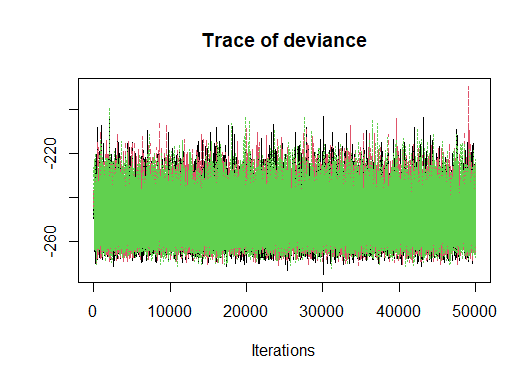 |
|  | Non-perturbed trials (block 1): EEG results | 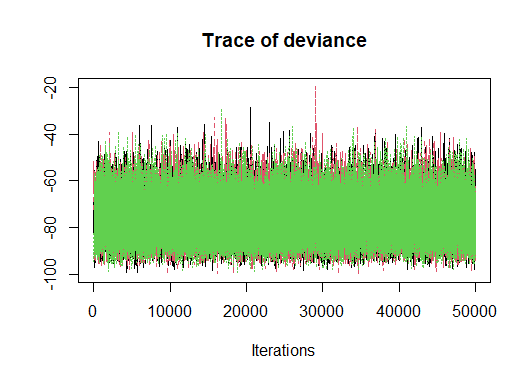 |
|  | Non-perturbed trials (mixed block): behavioral results | 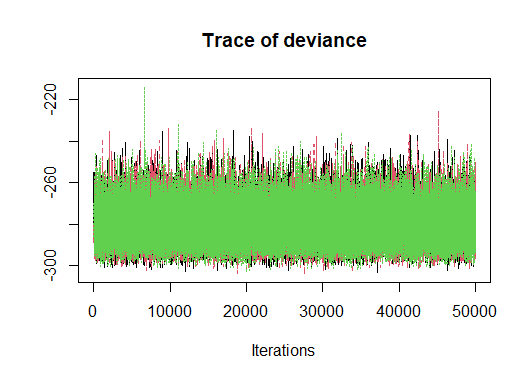 |
|  | Non-perturbed trials (mixed block): EEG results | 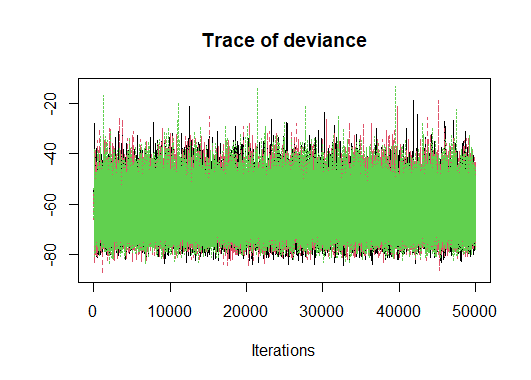 |
|  | Perturbed trials: behavioral results | 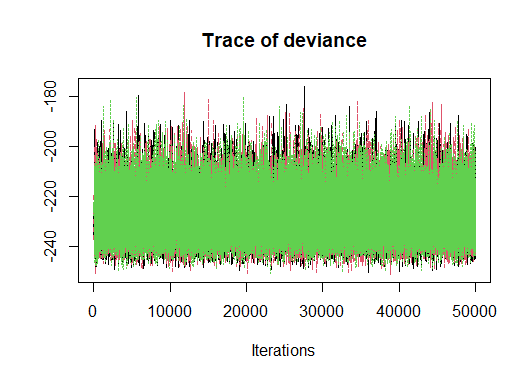 |
|  | Perturbed trials: EEG results | 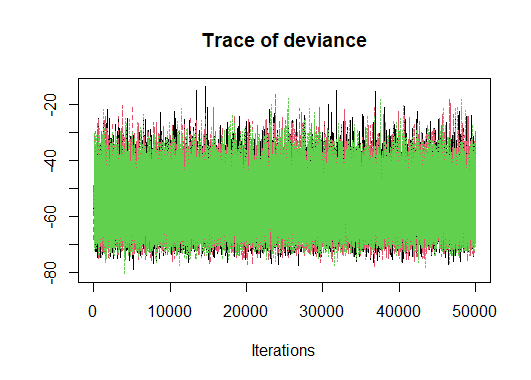 |
| Table S2 – convergence diagnostics. For each analysis, we ran 3 MCMC chains with 105,000 iterations each, discarding the first 5,000 as burn-in and applying a thinning interval of 2. The deviance trace plots show substantial overlap among the three chains, with no visible drift and a relatively constant spread, indicating a good mixing and stationarity and suggesting satisfactory convergence of the MCMC algorithm. | | |

Supplementary Material S5: Results before TMS – Scatter plots

| Behavioral data before intervention | Non-perturbed (mixed blocks) vs. Perturbed (mixed blocks) | 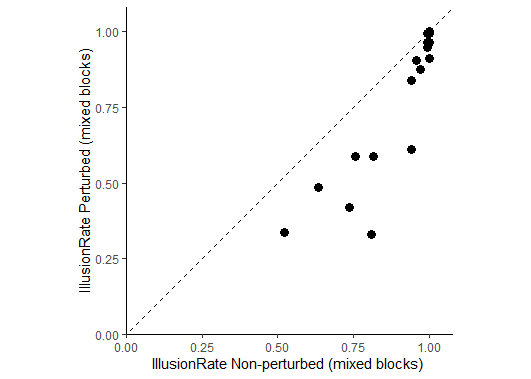  [N=19; OR = 0.38, CI95%: 0.18-0.68, *Pr*(perturbed (mixed blocks) > non-perturbed (mixed blocks) = 0.001))] |
| --- | --- | --- |
|  | Non-perturbed (first block) vs. Perturbed (mixed blocks) | 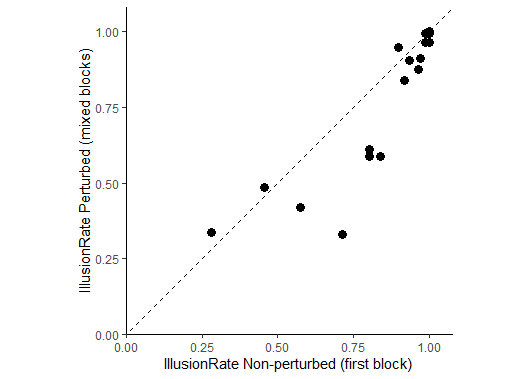  [N=19; OR = 0.46, CI95%: 0.22-0.82, *Pr*(perturbed (mixed blocks) > non-perturbed (block 1)) = 0.006] |
|  | Non-perturbed (first block) vs. Non-perturbed (mixed blocks) | 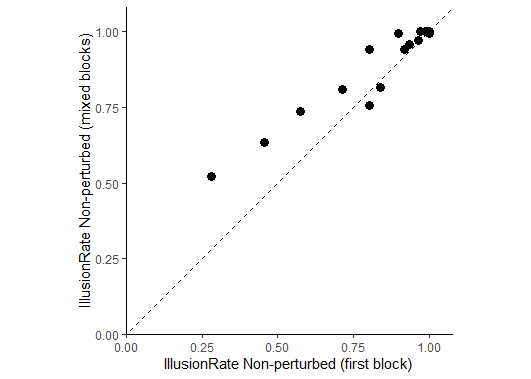  [N=19; *Pr*(non-perturbed (mixed blocks) > non-perturbed (block 1) ) = 0.7)] |
| EEG data before intervention | Non-perturbed (mixed blocks) vs. Perturbed (mixed blocks) | 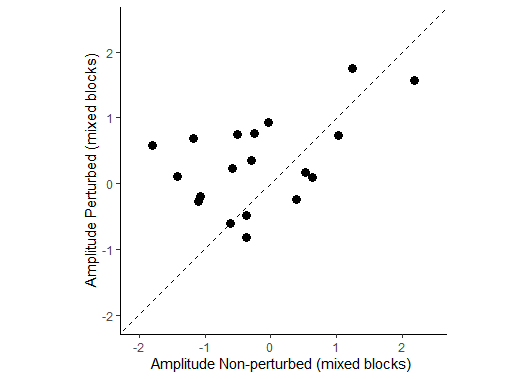  [N=19; OR = 5.5, CI95%: 2.89-9.59, *Pr*(perturbed (mixed blocks) > non-perturbed (mixed blocks) > 0.99)] |
|  | Non-perturbed (first block) vs. Perturbed (mixed blocks) | 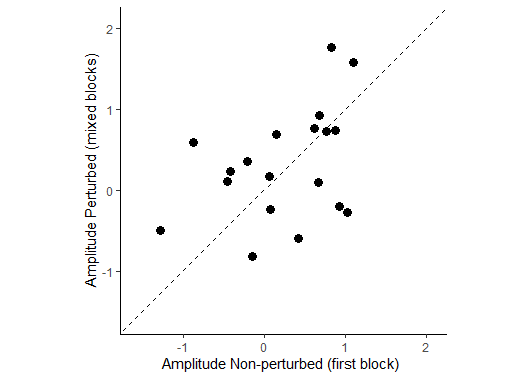  [N=19; OR = 2.6, CI95%: 1.36-4.6, *Pr*(perturbed (mixed blocks) > non-perturbed (first block)) = 0.99] |
|  | Non-perturbed (first block) vs. Non-perturbed (mixed blocks) | 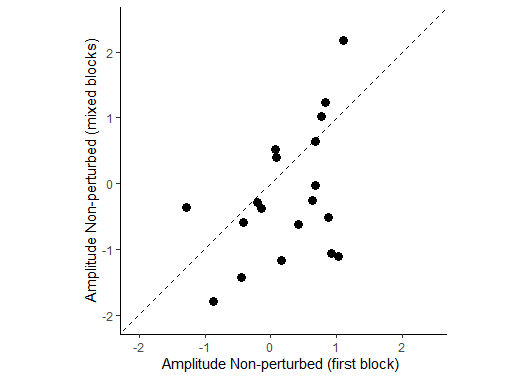  [N=19; OR = 0.5, CI95%: 0.27-0.83, *Pr*(non-perturbed (mixed blocks) > non-perturbed (first block)) = 0.004] |
| Table S3 – Scatter plots related to the main effects for the results before the intervention and data presented in Fig. 3. Scatter plots show contrasts between conditions and each black dot represents one participant. Below the scatter plots the respective statistical results can be found. | | |

Supplementary Material S6: Influences of TMS interventions on responses to the illusion

*S6.1 Non-perturbed trials of the first block*

Here, we considered only non-perturbed trials of the first block, see Fig. S3 a). We found no influence of *Intervention* (*Pr*(verum TMS > sham TMS) = 0.34), and no effect of time (*Pr*(after > before) = 0.47). Further, we found no interaction effect (*Pr* = 0.15). Regarding the two groups, we found no effect of *Session Order* (*Pr*(verum-sham > sham-verum) = 0.09), and no meaningful interactions, despite the graph suggesting otherwise (*Pr*s between 0.3-0.85).

The EEG results showed no effect of *Intervention* (*Pr*(verum TMS > sham TMS) = 0.72) and no effect for *Time Point* (*Pr*(after > before) = 0.12). We found a marginal interaction effect (*Pr* = 0.952), indicating no difference between verum and sham TMS before the intervention (Pr(verum TMS before > sham TMS before) = 0.72), but larger amplitudes for verum TMS than for sham TMS after the intervention (OR = 1.9, CI95%: 1.2-2.8, *Pr*(verum TMS after > sham TMS after) = 0.99), see Fig. S3 b). When considering *Session Order*, we did not find any clearly meaningful effects nor interactions.

| a) Behavioral data for non-perturbed trials (block 1)  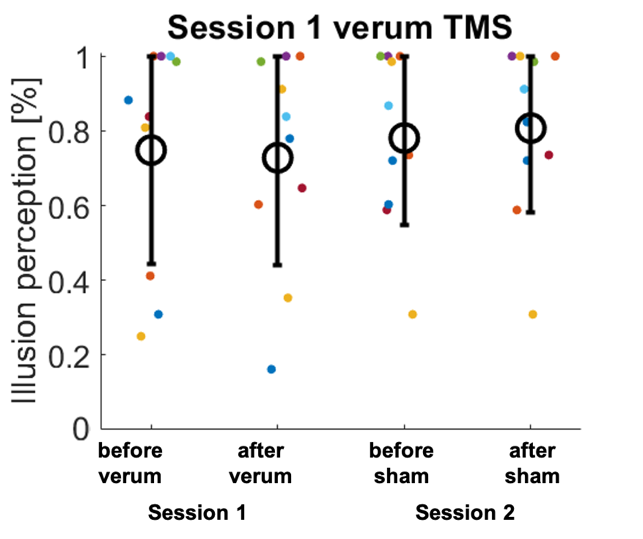 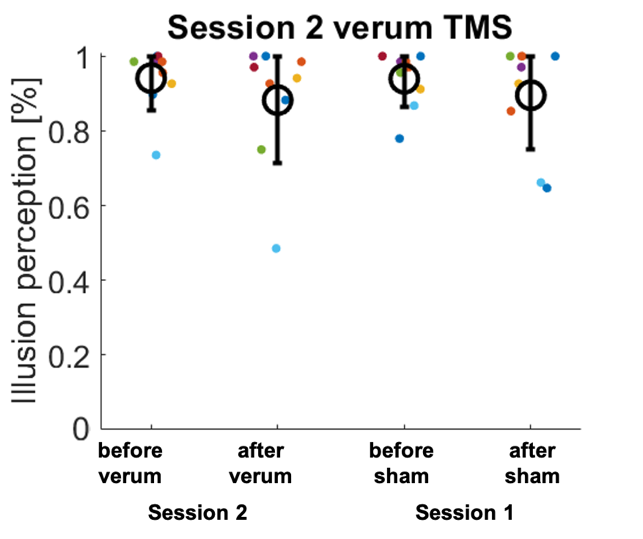 | |
| --- | --- |
| b) EEG data for non-perturbed trials (block 1)    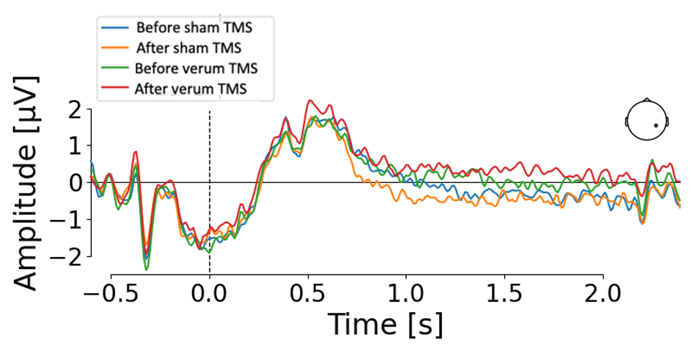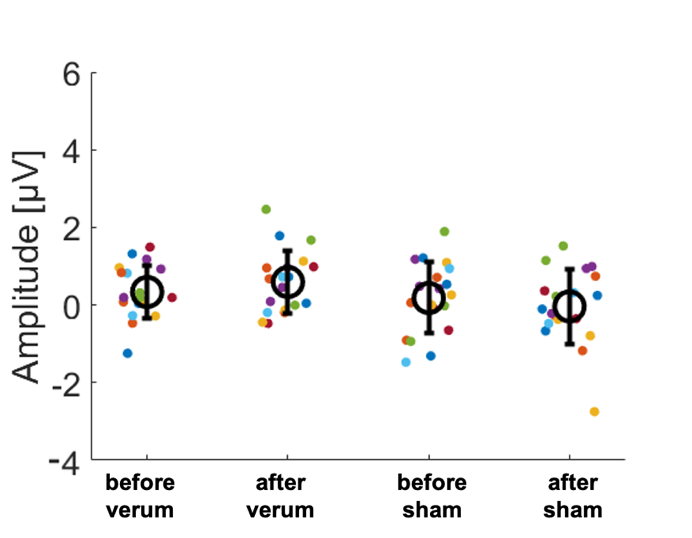 | |
| Figure S3 – Non-perturbed trials of the first block – the influence of *Time Point* and type of *Intervention.* In a) behavioral data, the illusion rate marginally decreased from before to after verum TMS, but stayed the same for sham TMS. On the left, the group receiving verum TMS on session 1 is depicted, which shows marginally smaller illusion rates than the group receiving verum TMS on session 2, depicted on the right. In b) EEG data, we depicted grand mean ERP data on the left, while on the right individual data is depicted. Small colored circles represent individual participants’ data, large empty black circles represent the mean and the error bars the standard deviation. |  |

*S6.2 Perturbed trials – EEG results*

|  | Factor | OR | CI95% | *Pr* | |
| --- | --- | --- | --- | --- | --- |
| Main factors | ***Intervention*** | **0.19** | **0.09-0.37** | **Verum TMS > sham TMS < 0.001** | |
|  | *Time Point* | 0.58 | 0.27-1.1 | After > before = 0.04 | |
|  | ***Session Order*** | **0.44** | **0.18-0.89** | **Verum-sham > sham-verum = 0.012** | |
| Interactions | ***Intervention * Time Point*** | **8.23** | **2.7-20** | **> 0.999** | |
|  | ***Intervention * Session Order*** | **7.77** | **2.61-18.27** | **> 0.999** | |
|  | *Time Point * Session Order* | 1.77 | 0.6-4.1 | 0.82 | |
|  | ***Intervention * Time Point * Session Order*** | **0.27** | **0.06-0.82** | **0.012** | |
| Table S4 – Main and interaction effects of the univariate Bayesian analyses conducted on the perturbed trials including the factors *Intervention* (verum TMS, sham TMS), *Time Point* (before intervention, after intervention), and *Session Order* (group verum-sham, group sham-verum). Meaningful effects are marked in bold. | | | | |  |

Investigating EEG amplitudes in response to the perturbed trials, we found several main effects and interactions. The table S4 details the results of this analysis. The meaningful interaction between *Intervention* and *Time Point* as well as the meaningful three-way interaction are described in detail in the main manuscript. Further, the pairwise comparisons for the meaningful three-way interaction were traced back to 13 meaningful pairwise comparisons that are listed below.

Meaningful comparisons for the group sham-verum:

- OR = 4.2, CI95%: 1.95-8.02, *Pr*(after intervention, verum TMS, sham-verum > before intervention, verum TMS, sham-verum) = 0.99
- OR = 0.19, CI95%: 0.09-0.37, *Pr*(before intervention, verum TMS, sham-verum > before intervention, sham TMS, sham verum) < 0.001

Meaningful comparisons for the group verum-sham:

- OR = 0.19, CI95%: 0.09-0.37, *Pr*(before intervention, verum TMS, verum-sham > before intervention, sham TMS, verum-sham) < 0.001
- OR = 0.33, CI95%: 0.09-0.89, *Pr*(after intervention, verum TMS, verum-sham > after intervention sham TMS, verum-sham) = 0.016
- OR = 0.2, CI95%: 0.03-0.69, *Pr*(after intervention, verum TMS, verum-sham > before intervention, sham TMS, verum-sham) = 0.007

Meaningful comparisons between groups:

- OR = 0.44, CI95%: 0.18-0.89, *Pr*(before intervention sham TMS, verum-sham > before intervention sham TMS, sham-verum) = 0.012
- OR = 0.44, CI95%: 0.18-0.89, *Pr*(before intervention verum TMS, verum-sham > before intervention verum TMS, sham-verum) = 0.012
- OR = 0.44, CI95%: 0.18-0.89, *Pr*(after intervention, sham TMS, verum-sham > after intervention, sham TMS, sham-verum) = 0.012
- OR = 0.14, CI95%: 0.014-0.54, *Pr*(after intervention, verum TMS, verum-sham > after intervention, verum TMS, sham-verum) = 0.005
- OR = 0.09, CI95%: 0.02-0.26, *Pr*(before intervention, verum TMS, verum-sham > before intervention, sham TMS, sham-verum) < 0.001
- OR = 0.16, CI95%: 0.02-0.62, *Pr*(after intervention, verum TMS, verum-sham > after intervention, sham TMS, sham-verum) = 0.006
- OR = 0,12, CI95%: 0.007-0.49, *Pr*(after intervention, verum TMS, verum-sham > before intervention, sham TMS, sham-verum) = 0.004
- OR = 0.27, CI95%: 0.06-0.77, *Pr*(after intervention, sham TMS, verum-sham > before intervention, sham TMS, sham-verum) = 0.009

*S6.3 Trial-to-trial effects:*

It is widely known that previous trials influence the perception of a current trial. In this analysis step, we investigated the influence of the perturbation level of a previous trial N-1 on the illusion perception rate of a current trial N, separately for its stimulus type (non-perturbed, perturbed). Analyses were conducted the same way as described in the main manuscript, i.e., with the factors *Intervention* (verum and sham TMS), *Time Point* (before and after intervention), and *Session Order* (group sham-verum, group verum-sham), and with the additional factor previous *trial N-1* (no perturbation or perturbation, averaged over the previous trials’ contact durations).

We did not find any clearly meaningful influence nor interaction of the identity of trial N-1 for the behavioral data (all *Pr* ranging between 0.11– 0.86), neither for the non-perturbed trials nor the perturbed trials at trial N. Regarding EEG data, we did not find any meaningful effects nor interactions of the identity of trial N-1 for the non-perturbed trials at trial N (all *Pr* ranging between 0.16-0.85).

When analyzing the EEG data of perturbed trials at trial N, we neither found a meaningful main effect, nor any interaction including *Intervention* and *Time Point*. We did find some interactions with the other factors, which, however, do not contribute to understand the meaningful 3-way interaction for EEG data related to perturbed trials, as described in the last result chapter of the main manuscript (see also Fig. 5 b).

Investigating EEG amplitudes in response to perturbed trials at trial N in an analysis considering also the identity of trial N-1, we did not find any main effect of *trial N-1 (Pr* = 0.97), nor the critical interaction between the factors *trial N-1*, *Intervention*, and *Time Point* (*Pr* = 0.13), nor a meaningful 4-way interaction including *Session Order* (*Pr* = 0.87).

References

Jovanovic, L., Trichanh, M., Martin, B., & Giersch, A. (2023). Strong perceptual consequences of low-level visual predictions: A new illusion. *Cognition*, *230*, 105279. https://doi.org/10.1016/j.cognition.2022.105279
